# Draft Genome Sequences and Antimicrobial Profiles of Three Staphylococcus epidermidis Strains from Neonatal Blood Samples

**DOI:** 10.1128/MRA.00170-21

**Published:** 2021-04-01

**Authors:** Innocent Afeke, Ahmed Moustafa, Misa Hirose, Mareike Becker, Hauke Busch, Axel Kuenstner, Anke Faehnrich, Anthony S. Ablordey, Christoph Haertel, Kokou Hefoume Amega-Aho, Mohamed Tarek Badr, Jan Rupp, Saleh Ibrahim

**Affiliations:** a Luebeck Institute for Experimental Dermatology, University of Luebeck, Luebeck, Germany; b Department of Medical Laboratory Sciences, School of Allied Health Sciences, University of Health and Allied Sciences, Ho, Ghana; c Department of Biology, Bioinformatics, and Integrative Genomics Laboratory, American University in Cairo, New Cairo, Egypt; d Department of Bacteriology, Noguchi Memorial Institute for Medical Research, University of Ghana, Accra, Ghana; e Department of Pediatrics, Wuerzburg University Clinic, University of Wuerzburg, Wuerzburg, Germany; f Department of Pediatrics, School of Medicine, University of Health and Allied Sciences, Ho, Ghana; g Institute of Medical Microbiology and Hygiene, Medical Center-University of Freiburg, Faculty of Medicine, Freiburg, Germany; h Clinic for Infectiology and Microbiology, University of Luebeck, Luebeck, Germany; University of Maryland School of Medicine

## Abstract

Data on molecular characterization of coagulase-negative staphylococci causing neonatal sepsis in low-income countries are highly limited. This report highlights the isolation of three Staphylococcus epidermidis non-genome assembly strains (NGASs) from blood samples from neonates with unknown transmission sources. Pathogenic factors and sources of transmission of these strains warrant further investigation.

## Abstract (precis)

Data on molecular characterization of coagulase-negative staphylococci causing neonatal sepsis in low-income countries are highly limited. This report highlights the isolation of three Staphylococcus epidermidis strains, NGASs, from blood samples from neonates, with unknown transmission sources. Pathogenic factors and sources of transmission of these strains warrant further investigation.

## ANNOUNCEMENT

We report the draft genome sequences of three Staphylococcus epidermidis non-genome assembly strains (NGASs) cultivated from blood samples obtained from neonates at the Ho Teaching Hospital (HTH) in Ghana. S. epidermidis is a coagulase-negative staphylococcus (CoNS), a permanent member of the human microbiota that commonly colonizes the skin and mucous membranes (1). S. epidermidis has become an important opportunistic pathogen among vulnerable patients, and it is reported to be a leading causative agent of nosocomial infections (2). S. epidermidis is the most prevalent CoNS commonly isolated from bloodstream infections in neonatal intensive care units (NICUs) (3). We have isolated, sequenced, and assembled three S. epidermidis NGASs from neonatal blood samples. Molecular typing of the *tuf* gene revealed them (of 74 isolates cultured from the HTH environment) to be peculiar strains isolated from blood culture samples; they had the highest levels of identity to a strain deposited in GenBank (accession number LR735440.1). Their transmission source was unclear, which prompted us to study these strains in more detail and to sequence their whole genomes. The Research Ethics Committee of the University of Health and Allied Sciences (Ho, Ghana) reviewed and approved this study (protocol UHAS-REC/A.2[1]17-18). Written approval was obtained from the HTH to use the facility for the study.

Blood samples were cultured using a Bactec 9050 system from March to June 2018. Positive culture bottles were subcultured on 5 to 10% sheep blood agar, harvested, and stored (in glycerol broth) at −20°C for 1 month before being shipped to Germany. Isolates were revived on blood agar plates incubated at 37°C for 24 h. Isolates were identified with a matrix-assisted laser desorption ionization–time of flight (MALDI-TOF) Biotyper G (Bruker Daltonics, Billerica, MA, USA). The MICs of 17 antibiotics were determined with a Vitek 2 system (bioMérieux, Durham, NC, USA). Genomic DNA was extracted using the DNeasy blood and tissue kit (Qiagen, Germany). Novogene (UK) Ltd. performed whole-genome sequencing. Library preparation was performed with the NEBNext Ultra DNA library preparation kit (New England Biolabs, Ipswich, MA, USA) according to the manufacturer's directions. The genomic DNA was randomly fragmented to a size of 350 bp by shearing. DNA fragments were end polished, A tailed, and ligated with the NEBNext adapter for Illumina sequencing and were further PCR enriched with P5 and indexed P7 oligonucleotides. The PCR products were purified (AMPure XP system), and the resulting libraries were analyzed for size distribution with an Agilent 2100 Bioanalyzer and quantified using real-time PCR. Library validation was performed on a Fragment Analyzer system (Agilent Technologies, CA) to control library quality, and quantification was performed using quantitative PCR via an ABI QuantStudio 12K Flex system (Thermo Fisher Scientific). Whole-genome sequencing was performed with an Illumina NovaSeq 6000 system to generate 150-bp paired-end reads. The genomes were sequenced at a minimum coverage of 100×. FASTQ files were processed to remove adaptor sequences, to trim low-quality ends, and to remove short reads using fastp v0.20.0 (3). To validate the isolates’ purity, the isolates were taxonomically classified on the basis of the sequencing reads using Kraken v2 (4) with the default taxonomy database. Sequencing reads were assembled using MEGAHIT v1.2.9 (5). Gene prediction and functional annotation were performed using Prokka v1.14.6 (6) with the BLAST, Pfam, and NCBI databases. The aforementioned software packages were used with default parameters.

A multilocus sequence typing (MLST) online search (https://cge.cbs.dtu.dk/services/MLST-2.0) was performed as described previously (7). Antibiotic resistance genes were searched for with the CARD Resistance Gene Identifier (https://card.mcmaster.ca/analyze/rgi) and IHU-Méditerranée Infection (https://ifr48.timone.univ-mrs.fr/blast/arg-annot_v6.html) websites. The staphylococcal cassette chromosome *mec* (SCC*mec*) type was determined with SCCmecFinder v1.2 (www.cge.cbs.dtu.dk/services/SCCmecFinder). Table 1 summarizes the results.

**TABLE 1:** Genome assemblies and antimicrobial susceptibility patterns of three Staphylococcus epidermis strains, HESN038B, HESN074B, and hHESN103B

Parameter	Data for strain^*b*^:		
	HESN038B	HESN074B	hHESN103B
MIC (mg/liter) (susceptibility category)^*a*^
Cefoxitin	2 (negative)	2 (negative)	>8 (positive)
Oxacillin	0.25 (NA)	2 (NA)	>2 (NA)
Erythromycin	0.25 (S)	0.5 (S)	>2 (R)
Clindamycin	0.25 (S)	0.25 (S)	>1 (R)
Amikacin	4 (S)	8 (S)	8 (S)
Ampicillin	2 (NA)	2 (NA)	>8 (NA)
Gentamicin	1 (S)	>4 (R)	>4 (R)
Tetracycline	0.5 (S)	0.5 (S)	>2 (R)
Fosfomycin with G6P^*c*^	16 (S)	16 (S)	16 (S)
Ciprofloxacin	0.25 (S)	0.25 (S)	4 (S)
Rifampin	0.25 (S)	0.25 (S)	0.25 (S)
Trimethoprim-sulfamethoxazole	4/76 (S)	>4/76 (R)	>4/76 (R)
Tobramycin	1 (S)	>4 (R)	>4 (R)
Penicillin G	0.0625 (NA)	>0.25 (NA)	>0.25 (NA)
Teicoplanin	1 (S)	1 (S)	1 (S)
Vancomycin	1 (S)	0.5 (S)	1 (S)
Mupirocin	1 (S)	1 (S)	1 (S)
Resistance genes	*dfrC*, *norA*, *fosB*, *gyrB*, IS*1272*	*dfrC*, *norA*, *fusB*, *far1*, *gyrB*, (AGly)*apH*-*stph*, *mecA*	*dfrC*, *dfrG*, *norA*, *gyrB*, IS*1272*
Genome size (bp)	2,682,678	2,439,254	2,431,156
No. of contigs	115	30	25
Size of largest contig (bp)	297,254	335,917	569,583
No. of reads	5,934,913	6,679,884	5,798,804
*N*_50_ (bp)	78,190	148,419	233,414
GC content (%)	31.8	32.01	32
No. of rRNAs	8	8	8
No. of coding sequences	2,508	2,238	2,233
No. of tRNAs	51	52	49
No. of transfer-messenger RNAs	0	1	1
MLST sequence type	Unknown	Unknown	226
SCC*mec* type	None	V (5C2&5)	None
BioProject accession no.	PRJNA668279	PRJNA668279	PRJNA668279
GenBank accession no.	JADCSK000000000	JADCSI000000000.1	JADCSS000000000.1
BioSample accession no.	SAMN16402351	SAMN16402349	SAMN16402359
SRA accession no.	SRR13236751	SRR13236760	SRR13236759

a The MIC results were interpreted according to 2018 European Committee on Antimicrobial Susceptibility Testing (EUCAST) clinical breakpoints (https://www.eucast.org/clinical_breakpoints).

b R, resistant; S, susceptible; NA, not applicable (no breakpoint available); V (5C2&5), type V SCC*mec* combination of the *ccr* gene complex (type 1) and *mec* gene complex class 2.

c G6P, glucose 6-phosphate.

### Data availability.

This whole-genome shotgun project has been deposited in DDBJ/ENA/GenBank under the accession numbers listed in Table 1.
